# Using a Short Food Frequency Questionnaire to Evaluate Macronutrients, Fiber, Phosphorus, Potassium, and Calcium in Adults with Stages 3–5 Chronic Kidney Disease

**DOI:** 10.3390/ijerph191911998

**Published:** 2022-09-22

**Authors:** Meng-Chuan Huang, Szu-Chun Hung, Tsen-Hua Tai, Ting-Yun Lin, Chiao-I Chang, Chih-Cheng Hsu

**Affiliations:** 1 Department of Nutrition and Dietetics, Kaohsiung Medical University Hospital, Kaohsiung Medical University, Kaohsiung 80708, Taiwan; 2 Department of Public Health and Environmental Medicine, School of Medicine, College of Medicine, Kaohsiung Medical University, Kaohsiung 80708, Taiwan; 3 Division of Nephrology, Taipei Tzu Chi Hospital, Buddhist Tzu Chi Medical Foundation, School of Medicine, Tzu Chi University, Hualien 97002, Taiwan; 4 Institute of Population Health Sciences, National Health Research Institutes, Zhunan, Miaoli County 35053, Taiwan; 5 Department of Health Services Administration, China Medical University, Taichung 40402, Taiwan; 6 Department of Family Medicine, Min-Sheng General Hospital, Taoyuan 33044, Taiwan

**Keywords:** chronic kidney disease, dietary record, short food frequency questionnaire, nutrition assessment

## Abstract

The progression of chronic kidney disease (CKD) can be directly or indirectly accelerated by a poor diet and the diet’s influence on risk factors for this disease. There have been no food frequency questionnaires (FFQs) developed for the assessment of diet in patients with CKD in Taiwan. This study analyzed the validity of a short FFQ (SFFQ) with 42 items for estimating patient intake of macronutrients, fiber, phosphorus, potassium, and calcium against 3-day dietary records (3-day DRs) in Taiwanese patients with stages 3–5 CKD. In an interview, 107 participants with the help of a dietician filled out an SFFQ and reviewed a 3-day DR the patients had filled out prior to the interview. Partial Pearson correlation coefficients between SFFQ and 3-day DR were 0.722, 0.619, 0.593, 0.572, 0.450, 0.611 and 0.410 for protein, fat, carbohydrate, fiber, phosphorus, potassium, and calcium, respectively, after adjusting for energy intake. Cross-classification analysis revealed 63.5–83.2% similarity in cross-tool estimated intakes of macronutrients, fiber, phosphorus, potassium, and calcium in the same quartiles or adjacent ones. Bland–Altman plots revealed good agreement between the two tools along different intake levels. In conclusion, the newly developed SFFQ had moderate relative validity in estimating the usual intake of key nutrients related to nutrition management of patients with late-stage CKD, suggesting it can be used to assess dietary intakes in a population with CKD, especially in those residing in an Asian region.

## 1. Introduction

Chronic kidney disease (CKD) has been associated with high risk of death and progress to end-stage renal disease (ESRD), requiring dialysis or transplantation [1], both expensive health procedures. The worldwide prevalence of CKD and CKD stages 3–5 are estimated to be 11–13% and 10.6%, respectively [2]. In 2008, the reported prevalence of CKD in Taiwan was similar (12%), with approximately 7% of the entire population suffering from CKD stages 3–5 [3]. Out of 52 countries, Taiwan has been recently reported to have the highest yearly prevalence and incidence of end-stage renal disease [4].

The definition of CKD includes all individuals with markers of kidney damage or those with an eGFR of less than 60 mL/min/1.73 m^2^ on at least two occasions 90 days apart (with or without markers of kidney damage) [5]. A poor diet may directly or indirectly accelerate CKD progression by contributing to the development of its major risk factors, including hypertension and type-2 diabetes [6]. In addition to some of the well-known symptoms (anemia, loss of appetite, fatigue, swelling of hands and legs, and shortness of breath), there are other more serious complications during the later stages of CKD. These complications include inflammation, CKD-mineral and bone disorder (osteodystrophy), cardiovascular disease, and infection as well as sodium, volume overload and hyperkalemia.. To delay the progression of CKD to ESRD, management of these symptoms through diet monitoring or effective nutritional intervention is essential [6,7]. 

Starting at CKD stage 3, patients become prone to developing metabolic disorders such as hyperparathyroidism, hyperphosphatemia, hyperkalemia, and metabolic acidosis, and thus it is important to regularly monitor calcium, phosphorus, potassium, and sodium intake [8]. Furthermore, it should be noted that approximately 40% of patients with CKD are also diagnosed with diabetes, being educated to avoid some specific nutrients (e.g., simple sugars and saturated fats) as required by patients with diabetes [9]. Thus, estimation of dietary intake is considered one of the most challenging tasks in clinical studies of patients with CKD.

Food intake assessment is integral to managing CKD. Food intake is usually estimated based on a subject’s self-perceived food intake over shorter periods of time using 24 h dietary recall tools and daily records of food intake as well as food frequency questionnaires (FFQs). FFQs are also routinely applied to rank subjects of a given population according to their nutrient intake over longer periods [10]. FFQs, previously tested in general populations, have been used to estimate specific nutrients or food intakes in patients with CKD in Australia [11], Korea [12], and Spain [13], with the number of questions on each FFQ ranging from 103 to 145. The FFQs developed in Taiwan have only been administered to healthy college students [14], older populations [15], general adults [16], and vegetarians [17]. No studies have been performed to develop FFQs for patients with CKD in clinical settings in Taiwan, despite the high prevalence of this serious disease there [3,4].

Food intake questionnaires need to demonstrate good validity and reliability and be simple and quick to administer in clinical settings where individual dietary counseling and planning is required [18]. Nutritionists and clinicians serving in units specializing in CKD or dialysis need to analyze critical nutrient intakes and identify dietary patterns associated with CKD progression. Ultimately, a quick valid dietary assessment tool could be invaluable to doctors and nurses, who do not usually assess diet, because they could use them to educate their patients on proper nutrition, encourage their adherence to a renal diet, and improve outcomes [19]. To date, there have been only two studies in France [20] and the USA [21] that have established FFQs and tested them in patients with CKD to examine diets or nutrients related to metabolism in CKD. Therefore, in this study, we aimed to evaluate the validity of a newly developed short-FFQ (SFFQ) comprising 42 questions for measuring specific nutrient intakes (energy, macronutrients, fiber, phosphorus, potassium, and calcium) in patients with CKD stages 3–5 in a hospital setting.

## 2. Materials and Methods

### 2.1. Study Design and Population

One hundred and seven adults with CKD stages 3–5 regularly attending an outpatient clinic belonging to the nephrology department at Tzu Chi Hospital were enrolled to join this cross-sectional study between February 2019 and February 2020. We excluded those < 20 years old, those who were pregnant, those who had an estimated glomerular filtration rate (eGFR) of 60 mL/min/1.73 m^2^ or higher, those who were receiving chronic dialysis or were scheduled to begin receiving chronic dialysis within the next three months, those who had polycystic kidney disease, those who had accepted immunosuppression therapy or any other type of immunotherapy to treat diagnosed primary renal disease or systemic vasculitis within the previous six months, those who had received transplantation of an organ or bone marrow, those who had received prescriptions for antibiotics during the previous three months as well as those with cirrhosis, HIV infections, cancer, pacemaker or metallic implants and amputees, because these patient characteristics suggested they may have other chronic diseases that would require a different diet, which would complicate our study. The protocol for this study was approved by the IRB at Taipei Tzu Chi Hospital (IRB No.07-XD-074). All participants signed written informed consent forms after the study details had been explained to them.

During recruitment, patients were instructed on ways to record the 3-day DR for three consecutive days, and days chosen needed to include 2 weekdays and one weekend day. After 1–2 weeks, patients brought back the 3-day DR to a dietitian who collected the patients’ 3-day DRs for later analysis. The subjects were interviewed by the same dietitian on the same day, at which time the FFQ was also administered. Before performing statistical analysis, we excluded those who had SFFQ-derived energy levels of less than 500 or greater than 7000 kcal/day [22] and those who had missing demographic and dietary data. For the present study, there was no subject with energy intake levels of less than 500 or greater than 7000 kcal/day. After exclusion, we were left with 107 patients whose data we used to evaluate the validity of the FFQ.

### 2.2. Collection of General Patient Characteristic Data

Anthropometric measurements including blood pressure, height, weight, waist and hip circumferences, and body mass index (BMI) were measured by registered nurses during outpatient department visits. Patients were asked to wear lightweight clothes and no shoes when weight was measured on standardized digital weight scales. Weight was rounded to the nearest 0.1 kg. In the measurement of height, the patients stood without shoes in front of standardized, wall-mounted height boards. Height was rounded to the nearest 0.5 cm. Body mass index (BMI) was classified into one of two categorical variables (normal weight 18.5–24 kg/m^2^, overweight > 24 kg/m^2^) based on cut-offs recommended by Taiwan’s Ministry of Health and Welfare [23]. We referenced cut-off points for waist circumference (WC), which were normal (<80 cm for women, <90 cm for men) and central obesity (WC ≥ 80 for women, ≥90 for men) using the standards by the International Diabetes Federation [24]. Anthropometric parameters and blood pressure were measured three times and averaged. A structured questionnaire was used to collect each patient’s age, gender, educational attainment, tobacco use (current smoker, ex-smoker, non-smoker), and ethanol use (drinker or non-drinker).

### 2.3. FFQ Assessment of Food Intakes

Generally, FFQs can be limited by underreporting and overreporting of intake of certain foods, lapses in memory, and the scarcity of trained interviewers [25]. Therefore, the interview for this study was conducted by a dietitian using a short (42-item) semi-quantitative paper-based FFQ or photograph-aided FFQ (SFFQ) designed to assess how frequently specific items from certain food groups were consumed, the size of serving portions, and other habits associated with eating. This photograph-aided SFFQ was created by two dietitians. The questionnaires along with photographs of food, common units (bowls/cups/spoons/bottles), and typical portions were organized into a photo-based booklet to support the administration of the SFFQ. We did not validate the photo-illustration booklet. It was a collection of photographs of food items covered in this SFFQ. It contained most food items with portion sizes established by Taiwan’s Health Promotion Administration, Ministry of Health and Welfare.

The participants were asked questions regarding the frequency with which they consumed certain food group items over the previous month leading up to the interview. There were nine frequencies, starting with “almost never” and ending with “four to six times per day.” The food groups assessed included bread and cereals, light- or dark-colored vegetables, root vegetables, packaged juices, fresh fruit, fish (marine or pond), meats (both red and white), soybean-derived food products, eggs, milk and dairy products, vegetables, as well as Chinese staple foods. Usual portion sizes consumed per day, week, or month were determined with the use of photographs. Per day and per month data were transformed into weekly equivalents. The intake of each nutrient per day was estimated by cross-referencing a food database established by Taiwan’s Food and Drug Administration; energy, macronutrients, and fiber were calculated as kcal or g per day.

The SFFQ we used was a modified and extended version of an FFQ that was previously developed and validated in patients with type-2 diabetes [22]. We organized food items into certain food groups, including white meat (goose, duck, chicken), red meat (beef, pork, lamb), animal organs, and fresh fruit (low, medium, or high potassium content, and bananas), in order to estimate the intake of energy, protein, phosphorus, potassium, and calcium as accurately as possible. The complete 42-item SFFQ does not include nutritional supplements and can be found in Supplementary Table S1. It clearly lists the 42 food items covered in the SFFQ.

### 2.4. Three-Day Dietary Record Assessment of Dietary Intakes

In order to improve the accuracy of the data collected by the 3-day DR, we trained our participants on how to record intake before they returned home to record their intake of foods for 3 days [18]. All participants were interviewed by a dietitian who instructed them on how to make a record of the food items they ate, the methods used to prepare the food, the times of day they ate, and the places where they ate. The dietitian also utilized actual commonly used utensils and containers of different sizes (cups and bowls, models of foods), and illustrations of food when they prepared the participants to fill out the 3-day DR. Additionally, dietitians encouraged the participants to use their mobile phones to take photos of foods as they recorded their diet. After being taught how to record diet, patients were told to record food consumption consecutively for two weekdays and one weekend day. Daily intakes were based on averages of daily intakes over 3 days. The same dietitian analyzed food nutrients in the foods the participants consumed using software developed for nutrient analysis (E-Kitchen Business Corporation, Taichung, Taiwan). Daily energy was expressed as kcal/day, and fiber, fat, protein, and carbohydrate intake were expressed as g/day, respectively, while calcium, phosphorus, and potassium intakes were expressed as mg/day, respectively.

### 2.5. Measurement of Clinical Parameters

We collected samples of overnight fasting venous blood at least eight to ten hours after the participant’s last meal. We also collected one-spot urine samples. All samples were stored in a −80 °C chamber before being transferred to a central laboratory at Taipei Tzu Chi Hospital. The laboratory is certified by Taiwan’s Accreditation Foundation. The laboratory used an auto-analyzer (Beckman Coulter, Fullerton, CA, USA) to measure cholesterol, high-density lipoprotein cholesterol (HDL-C), low-density lipoprotein cholesterol (LDL-C), triglyceride, plasma glucose, and uric acid levels, blood urea nitrogen (BUN) and creatinine. The auto-analyzer was also used to measure urine albumin and creatinine levels. High-performance liquid chromatography (Arkray, Inc., Kyoto, Japan) was used to measure hemoglobin A1c (HbA1c).

### 2.6. Statistical Analysis

FFQ- and 3-day DR-derived intake levels were calculated as mean ± SD or median analyzed into interquartile ranges (IQR). Statistical normality of macronutrients, fiber, energy, and mineral data were analyzed using a one-sample Kolmogorov–Smirnov test. To validate FFQ estimates, normal distribution of intake differences between FFQ and 3-day DR were assessed by paired t-test. Non-normal distributions of differences between estimates of the two tools were analyzed with the use of Wilcoxon’s sign ranked test. Log-transformation of data was performed to facilitate normalization. Intakes of energy and specific nutrients estimated by the two tools were correlated using Pearson correlation test. Partial correlations were based on data that had been adjusted for intake of energy, as previously applied to residual methods [26]. A correlation coefficient of 0.5–0.7 was considered acceptable [25]. We also analyzed the distribution of energy-adjusted nutrient intakes between the two assessment tools in quartiles, noting if they were classified as belonging to the same quartile, an adjacent quartile, one quartile apart, or extremely different quartiles. The percentage of subjects categorized into the lowest FFQ quartile and the percentage of those categorized into the highest 3-day DR quartile, or vice versa, were used to identify possible misclassifications. Agreement between nutrient intake data obtained by both tools was assessed using the Bland–Altman method [27]. IBM SPSS Statistics for Windows, version 22.0 (IBM Corp., Armonk, NY, USA) was used to perform all statistical operations. A *p*-value < 0.05 was considered significant.

## 3. Results

### 3.1. Patient Characteristics

Table 1 summarizes the characteristics of the 107 CKD patients enrolled in this study. The participants were mostly >65 years (58.9%), had high school educations or above (80.4%), and were non-smokers (61.7%). Mean BMI was 25.9 ± 4.6 kg/m^2^, and waist circumference 89.2 ± 12.1 cm. By distribution, CKD stages 3–5 were diagnosed in 30.8%, 39.3%, and 29.9% of the participants, respectively. Renal function indicators (eGFR, BUN, and serum creatinine), lipid parameters (triglycerides and total cholesterol) and blood pressure are also shown in Table 1.

### 3.2. FFQ Validity

Table 2 presents the mean daily intakes of energy, macronutrients, and micronutrients estimated by both FFQ and 3-day DR. With regard to the crude dietary data, Pearson correlation coefficients between the two assessment tools were 0.562 for energy, 0.573 for protein, 0.494 for fat, 0.426 for carbohydrate, 0.571 for fiber, 0.374 for potassium, 0.366 for phosphorus, and 0.385 for calcium. After adjustment for energy intake using the residual methods, there was improved correlation among the three macronutrients, three minerals, and fiber (all *p* < 0.001). As can be seen in Table 3, we cross-classified energy-adjusted nutrient intake levels into quartiles. The same quartile or adjacent quartile agreements ranged from 63.5% to 83.2%. Extreme quartile differences indicating misclassifications for these nutrients ranged from 0.9–11.2%.

**Figure 1:**
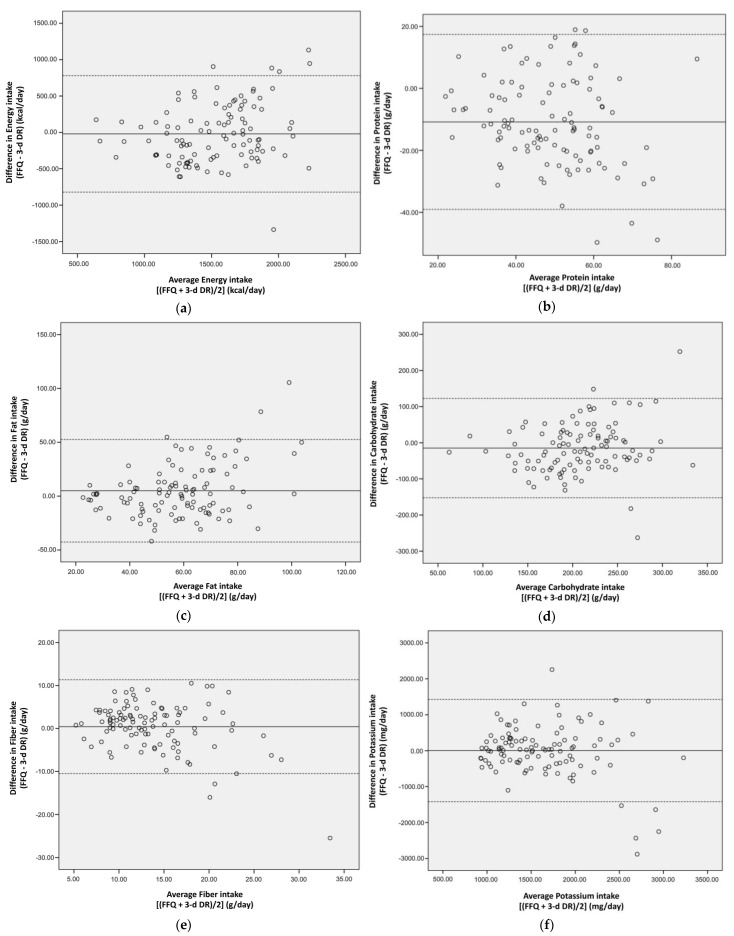

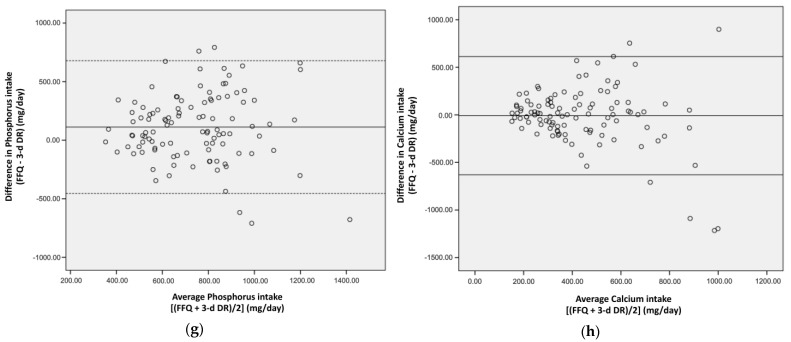
Bland–Altman plots demonstrating degree of agreement between the two assessment tools for daily intakes of (**a**) energy, (**b**) protein, (**c**) fat, (**d**) carbohydrate, (**e**) fiber, (**f**) potassium, (**g**) phosphorus, and (**h**) calcium in Taiwanese patients with chronic kidney disease (CKD). Solid lines indicate differences, and dashed lines indicate lower and upper limits (95%) of agreement.

As can be seen in Figure 1a–h Bland–Altman plots, there was adequate agreement between the SFFQ and 3-day DR assessments of daily intakes of energy, macronutrients, fiber, and the three minerals. Plots outside the lower and upper limits of agreement ranged from 2.8–6.5%. Considered by subheadings, these plots present a good description of our experimental results and their interpretation.

## 4. Discussion

For the present study, we developed an SFFQ customized for patients with late-stage CKD (CKD-SFFQ) and assessed its validity. The questionnaire was created to assess daily intakes of macronutrients, fiber, energy, phosphorus, potassium, and calcium. We found satisfactory validity overall, with the median proportion of participants grouped into the same quartiles or adjacent quartile by both the CKD-SFFQ and 3-day DR.

Several FFQ validation studies have employed short-term dietary measurements, including 24 h dietary recalls and diet records over their course of study [25]. The reference method we used to determine SFFQ validity in this study was the 3-day DR, which asks the subject to just recall the quantity of different foods consumed. FFQ is generally used to collect a few details regarding which foods were consumed, how they were prepared, and the portion sizes of these consumed. Therefore, quantification of intake may not be completely accurate. However, unlike dietary records or food recalls, FFQs are designed to characterize a subject’s daily dietary intakes. Most FFQs are filled out independently by a subject, and most are comparatively inexpensive to administer. Therefore, FFQs have been widely used to collect usual dietary intake data for large-scale epidemiologic studies despite their limitations, which include not being able to adequately quantify nutrient intakes [18]. Because of this limitation, it is important to compare any newly designed FFQ with a well-accepted reference method [28]. In a previous review, 22%, 25%, 26%, 6%, and 12% of the included validation studies employed multiple 24 h recalls, weighed records, food diaries (dietary records), diet history questionnaires, and other FFQs as reference measures, respectively [25]. Cross-test correlations for most foods and nutrients ranged from 0.40 to 0.70 [25]. The cross-test correlations we found for CKD-SFFQ for protein, carbohydrate, fat, fiber, phosphorus, potassium, and calcium intakes ranged from 0.41 to 0.72, suggesting an overall acceptable relative validity. 

In general, energy adjustment seems to diminish measurement errors associated with FFQs. Energy can be derived from most of the questionnaire items, and it can be used for adjustment in models estimating intake of other nutrients. The current study found improvements in the correlation coefficients after adjusting for energy. This particular improvement may come about as a result of energy adjustment to compensate for a substantial portion size error, based on reported experience with use of the Willett FFQ in many epidemiological studies [29]. Previous studies have developed long FFQs with questions ranging from 103–240 items [30,31,32], and some have been used to assess diets of patients with CKD [11,12]. We expected to find lower correlation coefficients for our FFQ compared to studies making use of questionnaires that cover a greater number of food items. It is reasonable to expect that an FFQ with more food items would provide a more accurate assessment of food intakes as well as nutrient intakes based on the contents of those foods. However, our study using a shorter FFQ found it to have similar or higher correlation coefficients than the longer FFQs used in the above studies [30,31,32]. Our study found similar or lower correlation coefficients compared to Barrat et al. (correlation coefficients = 0.59–0.80) [33], who also developed SFFQs. This difference may have resulted from our one-week focus and the proximity of our validity study and the administration of the FFQ. The differences between our results and the previous studies may also be partially due to cultural differences in diet.

The median proportion of our CKD patients were grouped into the same quartile or adjacent quartile regardless of assessment tool, with agreements ranging from 63.5% (protein) to 83.2% (fiber). This degree of agreement between the two tools’ quartile assignments of intake of both foods and nutrients of interest was acceptable and comparable with the 63–83% ranges reported by previous studies [20,32,33]. Our Bland–Altman plots displayed fair agreement between the two tools of estimation, across the range of intakes for macronutrients and important nutrients for dietary management of CKD. As shown in Figure 1, only 2.8% of the agreement values fell outside the lower and upper 95% limits (mean ± 1.96 standard deviation) for fat, 3.7% for carbohydrate and fiber, 4.7% for protein and phosphorus, 5.6% for energy and potassium, and 6.5% for calcium.

This study had several strengths. First, although FFQs exist, none of them have been tailored for Asians with CKD. Taiwan can be considered a representative Asian population. Second, the tool we developed can be administered more quickly than longer FFQs, and it has been validated against a widely accepted tool, the 3-day DR, possibly making the FFQ a valuable tool for clinicians treating patients with CKD in Taiwan. One of the strengths of our study is also a limitation. Our use of photos probably helped reduce recall bias in our study. However, most groups administering the questionnaire would not have access to these photos, so they might have trouble with recall bias, a common problem with FFQs. This study also has other limitations. One limitation was our small number of participants and limited number of food items queried. Another limitation was that our 3-day DR covered 3 days only, a period too short to capture the wide variety of foods or nutritional supplements consumed by our subjects over time. It has been suggested that such short-term assessments be repeated over a greater number of days and be averaged to obtain better estimates of usual intakes [18]. The correlation coefficients adjusted for energy intake were mostly above 0.5 for the nutrients surveyed in this study. Although FFQs with correlation coefficients greater than 0.5 for most nutrients can generally be considered reliable assessments of dietary intakes [34], future studies might want to investigate other biomarkers, including urinary urea-N (an indicator of protein intake), blood fatty acids (indicators of intake of foods containing various fats), and other biomarkers for micronutrients to determine acceptable correlation coefficients for them. Still another limitation was that almost all FFQs have inherent recall bias because individuals are asked to report what they remember about their intake, sometimes including intakes of foods long before the administration of the questionnaire. The accuracy of FFQs can also be affected by an individual’s intentional misreporting of their consumption of certain foods [35].

## 5. Conclusions

In conclusion, the newly developed SFFQ for this study, the first FFQ developed for CKD in Taiwan, has moderate relative validity in its estimation of the usual intakes of key nutrients, including macronutrients, fiber, phosphorus, potassium, and calcium in patients with late-stage CKD. The results of our validation study suggest that this SFFQ can be used as an acceptable tool for assessing dietary intakes of foods and nutrients as they relate to health status in this patient population, especially in Asia, where diets are similar.

## Figures and Tables

**Table 1:** Characteristics of 107 patients with chronic kidney disease (CKD).

Demographic and Clinical Characteristics	Overall
Gender	
Men	64 (59.8)
Women	43 (40.2)
Years old	
<65	44 (41.1)
≥65	63 (58.9)
CKD stage	
3	33 (30.8)
4	42 (39.3)
5	32 (29.9)
Education years	
<high school	21 (19.6)
>high school	86 (80.4)
Smoking status	
Non-smokers	66 (61.7)
Ex-smokers	29 (27.1)
Current smokers	12 (11.2)
Alcohol drinking	3 (2.8)
BMIs (kg/m^2^)	25.9 ± 4.6
<24.0	35 (32.7)
≥24.0	72 (67.3)
Waist circumferences (cm)	89.2 ± 12.1
Central obesity	
No	40 (37.4)
Yes	67 (62.6)
Systolic BP (mmHg)	135.8 ± 22.7
Diastolic BP (mmHg)	72.8 ± 9.0
Triglycerides (mg/dL)	145.1 ± 96.1
Total cholesterol (mg/dL)	157.0 ± 36.2
HbA1c (%)	6.2 ± 1.2
Blood urea nitrogen (mg/dL)	45.6 ± 23.2
Serum creatinine (mg/dL)	3.3 ± 2.0
eGFR (mL/min/1.73 m^2^)	24.4 ± 12.9

Abbreviations: HDL, high-density lipoprotein; LDL, low-density lipoprotein; HbA1c, glycated Hemoglobin. Data reported either as n (%) or mean ± SD.

**Table 2:** Correlations between data obtained by the food frequency questionnaire (FFQ) and 3-day dietary recall (3-day DR) (n = 107).

Nutrient	FFQ ^1^	3-Day DR ^1^	% Difference ^2^	*p* ^3,^ ^4^	Pearson Correlation Coefficient(r) ^5^	
					Crude ^6^	Adjusted ^7^
Energy (kcal/day)	1522.9 ± 435.7	1543.1 ± 350.3	0.7	0.273 ^3^	0.562 **	-
Protein (g/day)	43.5 ± 13.2	54.3 ± 15.9	−16.7	<0.001 ^3^	0.573 **	0.722 **
Fat (g/day)	60.6 ± 24.9	55.7 ± 17.5	14.5	0.157 ^3^	0.494 **	0.619 **
Carbohydrate (g/day)	200.7 ± 64.0	215.5 ± 53.9	−3.5	0.006 ^3^	0.426 **	0.593 **
Fiber (g/day)	13.1 (10.6–16.8)	12.5 (8.8–16.3)	15.3	0.077 ^4^	0.571 **	0.572 **
Potassium (mg/day)	1560.8 (1240.6–1910.5)	1525.0 (1119.9–1925.9)	11.8	0.574 ^4^	0.374 **	0.450 **
Phosphorus (mg/day)	774.3 (594.5–985.0)	632.4 (514.1–829.3)	27.1	<0.001 ^4^	0.366 **	0.611 **
Calcium (mg/day)	378.9 (252.3–595.5)	378.1 (244.7–561.3)	26.6	0.879 ^4^	0.385 **	0.410 **

^1^ Nutrient intakes derived from FFQ and 3-day DR reported as either mean ± SD or median categorized into interquartile ranges (IQR). ^2^ % difference = (FFQ) − (3-day DR)/3-day DR × 100. ^3^ Normality of intake differences between FFQ and 3-day DR were tested using paired *t* test. ^4^ Non-normal distribution of intake differences between the two assessment tools was tested using Wilcoxon’s signed ranked test. ^5^ Log-transformation of energy and nutrient data was first performed to normalize the distribution, followed by the calculation of correlation coefficients. ^6^ Pearson correlation coefficient was used to analyze crude data results. ^7^ Partial correlation coefficient was tested after adjusting for energy intake using the residual method. ** A *p*-value < 0.001 was considered significant.

**Table 3:** Cross-classification of short food frequency questionnaire- (FFQ-) and 3-day dietary recall- (3-day DR-) derived daily macronutrient intakes after adjusting for energy intake in 107 CKD patients.

Nutrient	Cross-Classification (%)				
	Same Quartile	Adjacent Quartile	Same or an Adjacent Quartile	One Quartile Apart	Extreme Quartile
Protein (g/day)	25.2	38.3	63.5	25.2	11.2
Fat (g/day)	31.8	35.5	67.3	21.5	11.2
Carbohydrate (g/day)	33.6	35.5	69.1	21.5	9.3
Fiber (g/day)	35.5	47.7	83.2	15.9	0.9
Potassium (mg/day)	36.4	38.3	74.7	21.5	3.7
Phosphorus (mg/day)	35.5	34.6	70.1	23.4	6.5
Calcium (mg/day)	32.7	44.9	77.6	19.6	2.8

Data are expressed as % of subjects grouped into same quartile, adjacent ones, a quartile apart, or extremely different quartiles.

## Data Availability

The data are not publicly accessible due to participant confidentiality associated with the ethical approval of this investigation.

## Supplementary Materials

The following supporting information can be downloaded at: https://www.mdpi.com/article/10.3390/ijerph191911998/s1.
