# Supplementary material for: Using a Short Food Frequency Questionnaire to Evaluate Macronutrients, Fiber, Phosphorus, Potassium, and Calcium in Adults with Stages 3–5 Chronic Kidney Disease
Source: Int J Environ Res Public Health. 2022 Sep 22;19(19):11998. doi: 10.3390/ijerph191911998 (PMC9565822; doi:10.3390/ijerph191911998)
Supplement: Supplementary file 1 [file ijerph-19-11998-s001.zip › ijerph-1881228-supplementary.pdf]

Manuscript Title: Using a Short Food Frequency Questionnaire to Evaluate Macronutrients, Fiber, Phosphorus, Potassium, and Calcium in Adults with Stages 3 – 5 Chronic Kidney Disease

Supplementary Table S1
42 item Short - Food frequency questionnaire (SFFQ) for chronic kidney disease (CKD)

**Food items**	**Frequency of food intake**	**Number of servings**
	**never or less than once<br>≤ 1 per month<br>2-3 per month<br>1-2 per week<br>3-4 per week<br>5-6 per week<br>1 per day<br>2-3 per day<br>4-6 per day**	**never or less than once<br><1 serving per time<br>1 servings per time<br>2 servings per time<br>3 servings per time<br>4 servings per time<br>5 servings per time<br>≧6 servings per time**
**1. Eggs (Chicken, duck, and bird eggs)**<br>One serving = an egg, five pigeon eggs, two egg white		
**2. Seafood (Prawn, calamari, crab, oyster, squid, and scallops)**<br>One serving = 4-6 prawns, 6 large clams, 22 small clams, 4 sliced of squids, 8 oysters, half a crab, 3 scallops, 2 cuttlefishes		
**3. Marine fish (Spanish mackerel, mackerel pike, salmon, ribbon fish, tilapia, and snapper, tuna)**<br>One serving = three fingers wide with a thickness of 1 cm= 2 fingers wide with a thickness of 2 cm		
**4. Freshwater fish (Milk fish/white mullet, tilapia, eel, and herring)**<br>One serving = three fingers wide with a thickness of 1 cm = 2 fingers wide with a thickness of 2 cm		
**5. How often are the above-mentioned fishes fried or deep-fried?**	**Approximately __________% fried**<br>□ 0% □ 1~20% □ 21~40% □ 41~61% □ 61~80%<br>□ 81~100 %	
**6. How often are the above-mentioned fishes grilled or steamed?**	**Approximately __________% grilled or steamed**<br>□ 0% □ 1~20% □ 21~40% □ 41~61% □ 61~80%<br>□ 81~100 %	
**7. Pork (pork ribs/chop/tendon, pork slices, and shoulder roast)**<br>One serving = three fingers wide with 1 cm thickness		
**8. Beef and lamb**<br>One serving = three fingers wide with 1 cm thickness		
**9. Chicken**<br>One serving = three fingers wide with 1 cm		

Food items	Frequency of food intake	Number of servings
	never or less than once<br>≤ 1 per month<br>2-3 per month<br>1-2 per week<br>3-4 per week<br>5-6 per week<br>1 per day<br>2-3 per day<br>4-6 per day	never or less than once<br><1 serving per time<br>1 servings per time<br>2 servings per time<br>3 servings per time<br>4 servings per time<br>5 servings per time<br>≧6 servings per time
thickness, 1/2 chicken drumstick, and 1 chicken wing		
**10. Duck and goose**<br>One serving = three fingers wide with 1 cm thickness, 1/2 duck/goose drumstick, and 1 duck/goose wing		
**11. How often are the above-mentioned meats fried or deep-fried?**	**Approximately __________% fried**<br>□ 0% □ 1~20% □ 21~40% □ 41~61% □ 61~80%<br>□ 81~100 %	
**12. Milk (Skimmed milk, light milk, and whole milk)**<br>One serving = 240 c.c. of milk, 3-4 tablespoons of whole milk powder, 3 tablespoons of light milk powder, 2.5 tablespoons of skimmed milk powder		
**13. Yogurt, yogurt drinks**<br>One serving = 240 c.c. yogurt drink, 200 c.c. yoghourt		
**14. Processed dairy products (Cheese slices, panna cotta, ice cream)**<br>One serving = 2 pieces of cheese slices, 1 cup of soft-serve ice cream, 120 c.c. of panna cotta		
**15. Soy products (fresh tofu, soy milk)**<br>One serving = 1/2 box soft fresh tofu , 1/2 block of firmed tofu (80 g), 240 c.c. of soy milk		
**16. Other soy products (Dried tofu, tofu skin, and fried bean curd)**<br>One serving = piece dried tofu, 1 slice tofu skin, and 2 blocks of fried bean curd		
**17. Dark-green colored vegetables (Broccoli, spinach, mushroom, sweet potato leaves, and Chinese green vegetables)**<br>One serving = 1/2 bowl cooked vegetables		
**18. Light-green colored vegetables (Cabbage, Chinese cabbage, Chinese radish, cauliflower, and water bamboo)**<br>One serving = 1/2 bowl cooked vegetables		
**19. Other vegetables (cucumber, bitter gourd, winter melon, loofah, tomato, eggplant,**		

Food items	Frequency of food intake	Number of servings
	**never or less than once**<br>**≤ 1 per month**<br>**2-3 per month**<br>**1-2 per week**<br>**3-4 per week**<br>**5-6 per week**<br>**1 per day**<br>**2-3 per day**<br>**4-6 per day**	**never or less than once**<br>**<1 serving per time**<br>**1 servings per time**<br>**2 servings per time**<br>**3 servings per time**<br>**4 servings per time**<br>**5 servings per time**<br>**≧6 servings per time**
**okra, green/sweet pepper, asparagus, carrot, onion, garlic, Chinese leek, and green onion, ginger)**<br>One serving = 1/2 bowl cooked vegetables		
**20. Mushroom (white mushroom, hericium, enoki mushroom, fungus, and king oyster mushroom)**<br>One serving = 1/2 bowl cooked vegetables		
**21. How often are the above-mentioned vegetables fried or using sauce?**	**Approximately __________% fried**<br>□ 0% □ 1~20% □ 21~40% □ 41~61% □ 61~80%<br>□ 81~100 %	
**22. Chinese staples - Rice**<br>One portion = 1 bowl rice, 2 bowl porridge		
**23. Chinese staples – Noodles**<br>One portion =1 bowl noodles, 2 bowls rice noodles		
**24. How often are the above Chinese staple foods fried? (Fried rice/noodles/rice noodles, biscuits fried dough sticks, dried noodles, braised pork rice)**	**Approximately __________% fried**<br>□ 0% □ 1~20% □ 21~40% □ 41~61% □ 61~80%<br>□ 81~100 %	
**25. Toast, bagel, bun, and steamed bun**<br>One portion = 2 slices of thick toast, 4 slices of regular toast, 4 buns, 1 bagel, 1 steamed bun		
**26. Whole wheat bread**<br>One portion = 2 slices of thick toast, 4 slices of regular toast		
**27. Bread with filling (soft roll, croissant, sweet bun, red bean bun, pineapple bun, and butter bread)**<br>One portion = 1 croissant, 1 sweet bun, 1 red bean bun, 1 pineapple bun, 1 butter bread		
**28. Root vegetables(Sweet potato, potato, taro, water caltrop, lotus root, Chinese yam, corn, and pumpkin)**<br>One serving = 4 hobs or 1/2 bowl, 1/2 cob of corn,		
**29. Low nitrogen staple foods (Rice noodle, bean noodle, pearl sago/tapioca, and tapioca starch)**<br>One Portion = 1 bowl of rice noodle/bean noodle,		

Food items	Frequency of food intake	Number of servings
	never or less than once<br>≤ 1 per month<br>2-3 per month<br>1-2 per week<br>3-4 per week<br>5-6 per week<br>1 per day<br>2-3 per day<br>4-6 per day	never or less than once<br><1 serving per time<br>1 servings per time<br>2 servings per time<br>3 servings per time<br>4 servings per time<br>5 servings per time<br>≧6 servings per time
4 tablespoon of tapioca pearls, 4 tablespoon tapioca starch		
**30. High potassium fruits (Cantaloupe, strawberry, kiwi, cherry tomato, and peach)**<br>One serving = 1 small-medium fruit (1 feast), 1/2-2/3 cup fresh fruits (80-120 g)		
**31. Moderate potassium fruits(California plums, sunkist, oranges, grapes, and guavas, papaya)**<br>One serving = 1 small-medium fruit (1 feast), 1/2-2/3 cup fresh fruits (80-120 g)		
**32. Low-potassium fruits (Wax apple, pineapple, mango, apple, watermelon, and pear)**<br>One serving = 1 small-medium fruit (1 feast), 1/2-2/3 cup fresh fruits (80-120 g)		
**33. Banana**<br>One serving = 1/2 medium banana		
**34. 100% fresh fruit juice**<br>One serving = 1/2 cup (about 120 c.c.)		
**35. 10% sweetened fruit juice (Bottled juice or fresh juice with sugar, and condensed juice)**<br>One serving = 1/2 cup (120-150 c.c.)		
**36. Cereal-based beverages**<br>One portion = 280-300 cc oat/cereal milk = 6 tablespoon oat = 4 tablespoon whole grain powder= 3 tablespoon pearl barley		
**37. Seeds and nuts (peanut, pine nuts, almond, pistachio, walnut, cashew nut, sesame, and pumpkin seeds)**<br>One serving = pine nuts 1 table spoon, 10 peanut, 6-8 almond, 15 pistachio, 2 walnut, 6-7 cashew nut, 50 melon seeds, 40 pumpkin seeds		
**38. Vegetable fats and oils (soybean oil, olive oil, safflower seed oil, canola oil, corn oil, palm oil, and coconut oil)**<br>One serving = 1 teaspoon		
**39. Animal fat and oils and Hydrogenated oils**		

Food items	Frequency of food intake	Number of servings
	never or less than once<br>≤ 1 per month<br>2-3 per month<br>1-2 per week<br>3-4 per week<br>5-6 per week<br>1 per day<br>2-3 per day<br>4-6 per day	never or less than once<br><1 serving per time<br>1 servings per time<br>2 servings per time<br>3 servings per time<br>4 servings per time<br>5 servings per time<br>≧6 servings per time
One serving = 2 teaspoon of butter/cream/lard, one teaspoon of hydrogenated oils		
**40. Hand-shaken beverages**<br>**if you answer question 40, please go on to answer questions 41, 42**		
**41. Cup size**	**□ small (360 ml) □ medium (500 ml) □ big (750 ml) □ extra-large (1000 ml)**	
**42. Sugar contents**	**□ sugar-free □ quarter sugar □ half sugar □ less sugar □ regular sugar**	
